# The effects of dietary fiber level on nutrient digestibility in growing pigs

**DOI:** 10.1186/2049-1891-4-17

**Published:** 2013-04-15

**Authors:** Wenjuan Zhang, Defa Li, Ling Liu, Jianjun Zang, Qiwu Duan, Wenjun Yang, Liying Zhang

**Affiliations:** 1 State Key Laboratory of Animal Nutrition, China Agricultural University, Beijing, 100193, P. R. China

**Keywords:** Growing pigs, Insoluble dietary fiber, Nutrient digestibility, Soluble dietary fiber, Total dietary fiber

## Abstract

The objective of this study was to investigate the effects of total dietary fiber level on nutrient digestibility and the relationship between apparent total tract digestibility of total dietary fiber, and soluble dietary fiber, insoluble dietary fiber and available energy. Sugar beet pulp was as the only fiber source. The experiment was designed as a 6 × 6 Latin square with an adaptation period of 7 d followed by a 5-d total collection of feces and urine. Feed intake tended to decrease (*P =*0.10) as total dietary fiber level increased. The apparent total tract digestibility of dry matter, crude protein and gross energy decreased (*P <*0.01) when total dietary fiber increased but the digestibility of soluble dietary fiber and insoluble dietary fiber increased (*P <*0.01). The digestible energy and metabolizable energy content of diets decreased (*P <*0.01) as the total dietary fiber increased.

## Background

Total dietary fiber (TDF) is the sum of the dietary carbohydrates that are resistant to digestion by mammalian enzymes in the small intestine but can be partially or completely fermented in the hindgut [1]. According to its solubility, TDF can be divided into soluble dietary fiber (SDF) and insoluble dietary fiber (IDF) [2]. Dietary fiber is a key factor determining nutrient utilization in the diet and more emphasis should be given to routine techniques that identify the nutritional and physiological “quality” of dietary fiber [3].

Serena et al. (2008) reported that SDF has a high water holding capacity, delays gastric emptying, slows the rate of nutrient absorption [4]. Most of SDF and partial of IDF are degraded by bacteria in either the small or large intestine [4-6]. Energy produced by microflora in the hindgut can satisfy up to 30% of the maintenance energy requirements of the pig [7]. In addition, IDF was found to decrease intestinal transit time, binds organic compounds and increases fecal bulk [8].

A minimum level of fiber has to be included in pig diets to support normal physiological activity in the digestive tract [9]. Mateos et al. (2006) suggested that young pigs may have a minimum requirement for a fiber level of 6% [10]. However, diets or ingredients with a high fiber content may negatively affect voluntary feed intake and nutrient digestibility in young pigs [11,12]. Understanding the effects of TDF level in the diet on nutrient digestibility and feed intake is critical for optimal swine production.

The effect of TDF on the digestibility of nutrients in the diet is controversial. Wilfart et al. (2007) added 0, 20 and 40% wheat bran to a wheat-barley-soybean meal diet and found that an increase of TDF significantly decreased the apparent total tract digestibility (ATTD) of dry matter, organic matter, crude protein and gross energy, but the ATTD of TDF was unaffected [12]. However, Urriola and Stein (2010) reported that the digestibility of dry matter, gross energy and TDF in diet with 30% distillers dried grains with soluble (DDGS) was significantly lower than a corn-soybean meal control diet fed to growing pigs [13]. Additionally, Bindelle et al. (2009) reported that when growing pigs were fed corn-soybean meal diets supplemented with sugar beet pulp at levels of 0, 10, 20 and 30%, the TDF level increased from 9.6 to 25.4% while the ATTD of dry matter, organic matter and crude protein linearly decreased but the ATTD of NDF was linearly increased [14]. Some of these differences may be due to differences in ATTD of TDF between the basal diet and the fiber sources.

The impact of fiber level on digestibility may differ with the properties of the fiber (i.e. soluble vs. insoluble) [15]. Sugar beet pulp is characterized by a high content of soluble fiber such as pectins and glucans [16]. However, there are no reports in the literature about the relationship between the type of fiber and its affects on nutrient digestibility. Therefore, in the present experiment, sugar beet pulp was added to diets to determine the effect of fiber type on nutrient digestibility and to study the relationship between the apparent total tract digestibility of TDF and SDF or IDF.

## Materials and methods

The Institutional Animal Care and Use Committee at China Agricultural University (Beijing, China) reviewed and approved the protocols used in this study.

### Experiment design and housing

Six healthy crossbred (Duroc × Landrace × Large White) barrows were allotted to a 6 × 6 Latin square design. The pigs (average initial body weight of 30.0 ± 1.8 kg) were individually housed in 1.2 m × 0.7 m × 0.96 m stainless steel metabolism cages in an environmentally controlled room (22 ± 2°C).

### Diets and feeding

Table 1 shows the nutrient content of the main ingredients used in this experiment. Six diets were formulated by replacing the basal diet with 0, 15, 25, 35, 45 or 55% sugar beet pulp (Table 2). The sugar beet pulp was ground to pass through a > 3.5 mm mesh screen before mixing into the diets. The protein level of diets was maintained at the same level by adjusting the amount of casein. The mineral, vitamin and amino acid premix was added to the diets at a level sufficient to meet or exceed the nutrient requirements of the National Research Council [17] for pigs weighing 20 to 50 kg.

**Table 1:** Chemical composition of rice starch, casein and sugar beet pulp (as-fed basis)

			**Nutrient value **^**a**^**, %**			
	**Moisture**	**Gross energy [MJ/kg]**	**Crude protein**	**TDF**	**SDF**	**IDF**
Rice starch	12.6	14.7				
Casein	8.2	21.6	82.4			
Sugar beet pulp	13.1	15.9	9.6	69.1	22.0	47.1

^a^Analyzed values. TDF = Total dietary fiber; SDF = Soluble dietary fiber; IDF = Insoluble dietary fiber.

**Table 2:** Ingredient composition, energy and nutrient levels of the diets (% as fed)

	**Sugar beet pulp, %**					
	**0.0**	**15.0**	**25.0**	**35.0**	**45.0**	**55.0**
Ingredients, %						
Rice starch	76.0	62.8	54.0	45.2	36.3	27.5
Casein	20.0	18.2	17.0	15.8	14.7	13.5
Sugar beet pulp	0.0	15.0	25.0	35.0	45.0	55.0
Vitamin and mineral premix ^a^	4.0	4.0	4.0	4.0	4.0	4.0
Energy and nutrient values ^b^						
Digestible energy, MJ/kg	14.7	14.8	14.2	14.0	13.6	13.1
Crude protein, %	17.5	17.3	17.3	17.4	17.3	17.3
Total dietary fiber, %	0.0	12.1	18.8	24.8	32.0	38.9
Soluble dietary fiber, %	0.0	4.2	6.0	8.0	10.1	12.2
Insoluble dietary fiber, %	0.0	7.9	12.8	16.8	21.9	26.7
Calcium, %	0.77	0.79	0.81	0.81	0.80	0.82
Phosphorus, %	0.50	0.55	0.54	0.57	0.54	0.55
	**Sugar beet pulp, %**					
	0.0	15.0	25.0	35.0	45.0	55.0
Amino acds, %						
Aspartic acid	1.28	1.26	1.13	1.26	1.14	1.34
Threonine	0.64	0.70	0.69	0.71	0.71	0.78
Serine	0.81	0.88	0.88	0.83	0.76	0.87
Glutamic acid	3.85	3.96	3.86	3.65	3.53	3.48
Proline	1.61	1.69	1.59	1.40	1.61	1.48
Glycine	0.42	0.37	0.35	0.42	0.41	0.50
Alanine	0.65	0.61	0.66	0.64	0.65	0.68
Valine	1.08	1.14	1.02	1.11	1.00	1.14
Isoleucine	0.89	0.90	0.85	0.86	0.82	0.85
Leucine	1.56	1.63	1.59	1.52	1.49	1.50
Tyrosine	0.82	0.80	0.72	0.76	0.72	0.77
Phenylalanine	0.92	0.92	0.90	0.88	0.85	0.84
Histidine	0.47	0.48	0.54	0.49	0.50	0.50
Lysine	1.55	1.60	1.54	1.58	1.46	1.58
Arginine	0.41	0.55	0.56	0.56	0.55	0.63
Methionine	0.44	0.46	0.47	0.47	0.47	0.48
Tryptophan	0.21	0.22	0.21	0.23	0.22	0.21

^a^ Supplied per kilogram of diet: vitamin A;6.0 KIU; vitamin D_3_; 2.4 KIU; vitamin E,21.6 IU; vitamin K, 2.0 mg; thiamine 1.0 mg; riboflavin 5.2 mg; pyridoxine 2.0 mg; vitamin B_12_ 0.01 mg; D-pantothenic acid 11.2 mg; niacin 22 mg; biotin 40 μg; folic acid 0.4 mg; 120 mg of Fe; 120 mg of Zn; 40.0 mg of Mn; 80 mg of Cu; 400 μg of I; and 240 μg of Se; 8.0 g of calcium; 0.4 g of phosphorus.

^b^ The nutrient levels are analyzed values. Digestible energy were calculated values.

The daily feed allowance was equivalent to 4% of body weight at the beginning of each period [18]. The allowance was divided into two equal parts and fed at 08:00 and 17:00 h. The diets were mixed with water in a ratio of 1:1 (Wt/Wt) before feeding. Water was available *ad libitum* through a drinking nipple. The pigs were weighed individually at the beginning of each period and the amount of feed supplied each period was recorded, as well as any feed refusals. Each experimental period consisted of a 7-d adaptation period followed by a 5-d collection of feces and urine. The collected urine was weighed and 10% of the daily urine volume was stored at -20°C. The collection of feces and urine were conducted according to the methods described by Song et al. (2003) [19]. Feces were collected immediately when the feces appeared in the metabolism cages, kept in plastic bags and stored at -20°C. Urine was collected into urine collection buckets that were placed under the metabolism cages. The buckets were emptied each afternoon and 50 mL of 6 mol/L HCl was added. At the end of the experiment, feces and urine samples were thawed and mixed within animal and diet, and a subsample was collected for chemical analysis. Fecal samples were dried in a forced air oven and ground through a 1-mm screen, and thoroughly mixed before a subsample was collected for chemical analysis.

### Chemical analyses

Diets and feces were analyzed for dry matter (AOAC method 930.15) [20] and crude protein (AOAC method 990.03) [20]. TDF and IDF were also determined (AOAC method 985.29) [20].The concentration of SDF in the diets was calculated as the difference between TDF and IDF. In addition, the diets were analyzed for calcium and total phosphorus (AOAC method 985.01) [20]. The gross energy in diets, feces, and urine were analyzed using an adiabatic oxygen bomb calorimeter (Parr Instruments, Moline, IL). The content of nitrogen in the urine was also analyzed (AOAC method 990.03) [20]. Amino acids in the feeds were determined by hydrolyzing the feed with 6 mol/L HCl for 24 h at 110°C (AOAC method 982.30 E) [20] and analyzed using a Hitachi L-8900 Amino Acid Analyzer (Tokyo, Japan). Methionine was determined as methionine sulfone after cold performic acid oxidation over night and hydrolyzing with 7.5 mol/L HCl for 24 h at 110°C.

### Statistical analysis

The data for effects of dietary sugar beet pulp on the apparent total tract digestibility (ATTD) of fiber, dry matter, protein, energy and the available energy of diets in growing pigs were subjected to an Analysis of Variance using PROC GLM of SAS (Statistical Analysis System 9.1, SAS Institute, Cary, NC, USA). Orthogonal polynomial contrasts were used to determine linear and quadratic effects of the TDF level on ATTD of energy, nutrients and the available energy of dies in growing pigs. Pig and period were random effects, and TDF level was considered a fixed effect. The PROC CORR and GLM of SAS were used to analyze the relationship between the ATTD of TDF and the ATTD of IDF or SDF. The model included dietary treatment and the residual mean square error was used as the error term. The means were separated using Duncan’s new multiple range test. The individual pig (n = 6 pigs/treatment group) served as the experimental unit. Results are reported as means plus standard errors with *P* < 0.05 defined as significant and *P* < 0.10 as indicative of a trend.

## Results

### The effects of sugar beet pulp on feed intake, fecal output, and the relationship between digestibility of TDF and IDF or SDF

**Figure 1:**
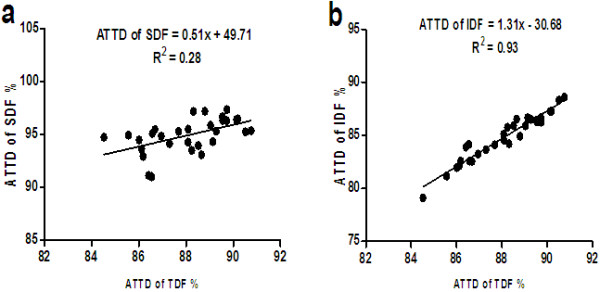
Relationship between the apparent total tract digestibility (ATTD) of totaldietary fiber (TDF) and the ATTD of soluble fiber (SDF) (a) and insoluble dietary fiber (IDF) (b) in sugar beet pulp fed to growing pigs (n = 30).

The effect of TDF on the digestibility of fibrous components is shown in Table 3. The feed intake of diets tended to decrease (*P =*0.10) as the TDF increased. As expected, the intake of TDF, SDF and IDF and the excretion of TDF and IDF increased (*P <*0.01) as TDF increased, but the excretion of SDF was unaffected by TDF. The output of feces increased (*P <*0.01) as the TDF increased. The ATTD of TDF, SDF and IDF increased (*P <*0.01) when TDF increased. The relationship between ATTD of SDF, IDF and TDF is shown in Figure 1. There was a good relationship between ATTD of TDF and IDF (r^2^ = 0.93), but a poor relationship between the ATTD of TDF and SDF (r^2^ = 0.28).

**Table 3:** Effects of dietary sugar beet pulp on the apparent total tract digestibility (ATTD) of fiber in growing pigs

**Items**	**Sugar beet pulp, %**						***P *Values**		
	**0.0**	**15.0**	**25.0**	**35.0**	**45.0**	**55.0**	**SEM**	**Linear**	**Quadratic**
Intake, g/d									
Feed intake	1,190.0	1,193.0	1,193.0	1,205.0	1,107.0	1,062.0	64.15	0.09	0.16
Total dietary fiber	0.0	124.0	206.4	292.8	344.3	404.6	23.86	<0.01	<0.01
Soluble dietary fiber	0.0	39.4	65.6	92.8	109.6	128.5	7.58	<0.01	<0.01
Insoluble dietary fiber	0.0	84.7	140.8	200.0	234.7	276.1	16.28	<0.01	<0.01
Excretion, g/d									
Feces, wet basis	135.0	194.9	317.0	409.7	415.2	571.5	19.60	<0.01	<0.01
Feces, dry matter basis	79.3	90.2	127.8	145.3	124.9	159.8	9.00	<0.01	<0.01
Total dietary fiber	0.0	29.9	29.3	35.6	36.4	43.2	3.34	<0.01	<0.01
Soluble dietary fiber	0.0	3.5	3.7	5.5	4.1	4.7	0.67	0.22	0.38
Insoluble dietary fiber	0.0	16.4	25.6	30.1	32.3	38.4	2.86	<0.01	<0.01
ATTD of fiber, %									
Total dietary fiber	0.0	86.2	86.9	88.1	89.7	89.5	0.45	<0.01	<0.01
Soluble dietary fiber	0.0	92.9	94.9	94.3	96.3	96.3	0.49	<0.01	<0.01
Insoluble dietary fiber	0.0	82.6	83.2	85.1	86.7	86.4	0.80	<0.01	<0.01

### The effects of sugar beet pulp on the digestibility of dry matter, crude protein and energy

The effect of TDF on the digestibility of dry matter, crude protein and energy is shown in Table 4. The gross energy intake tended to decrease (*P =*0.10) as TDF increased. In addition, the amounts of nitrogen and energy excreted from feces were increased (*P <*0.01) as dietary fiber level increased, while the amounts of energy excreted from urine was decreased (*P <*0.05). The ratio of urinary nitrogen to fecal nitrogen tended to decrease (*P =*0.10) as TDF increased although the amount of nitrogen excreted from urine was not affected (*P >*0.1) by the dietary fiber level. The digestibility of dry matter, crude protein and energy decreased (*P <*0.01) as TDF increased.

**Table 4:** Effects of dietary sugar beet pulp on the apparent total tract digestibility (ATTD) of dry matter, protein, energy and the available energy of diets in growing pigs

**Items**	**Sugar beet pulp, %**						***P *Values**		
	**0.0**	**15.0**	**25.0**	**35.0**	**45.0**	**55.0**	**SEM**	**Linear**	**Quadratic**
Intake									
Dry matter intake, g/d	1,088.0	1,095.0	1,091.8	1,083.0	1,022.3	968.3	56.96	0.46	0.26
Nitrogen intake, g/d	33.4	33.6	34.2	33.6	29.8	29.6	1.69	0.40	0.28
Gross energy intake, MJ/d	17.9	18.7	18.5	18.8	17.1	16.3	0.58	0.10	0.07
Excretion									
Fecal nitrogen, g/d	1.9	3.3	4.8	5.2	4.9	6.1	0.44	<0.01	<0.01
Urinary nitrogen, g/d	9.3	7.6	7.4	7.0	6.8	6.2	1.00	0.27	0.54
Urinary:fecal nitrogen ratio	4.9	2.3	1.6	1.4	2.0	1.1	0.13	0.10	0.17
Fecal energy, MJ/d	0.4	1.1	1.6	1.9	2.0	2.3	0.13	<0.01	<0.01
Urinary energy, MJ/d	0.9	0.7	0.6	0.6	0.4	0.4	0.11	<0.01	0.02
ATTD, %									
Dry matter	95.4	91.8	88.3	86.6	87.8	83.5	0.32	<0.01	<0.01
Nitrogen	94.6	90.0	85.9	84.4	83.7	80.0	1.00	<0.01	<0.01
Energy	97.8	94.5	91.4	89.9	88.4	86.3	0.47	<0.01	<0.01
Energy content of diets, MJ/kg									
Digestible energy	14.7	14.8	14.2	14.0	13.6	13.1	0.07	<0.01	0.06
Metabolizable energy	14.0	14.2	13.7	13.5	13.2	12.8	0.10	<0.01	0.71

### The effects of sugar beet pulp level on the available energy in the diet

The digestible energy (DE) and metabolizable energy (ME) content of the diets decreased (*P <*0.01) when the level of sugar beet pulp increased from 15.0 to 55.0% (Table 4). The correlation between the content of TDF and DE, ME of diets is shown in Table 5. The content of TDF had a negative correlation with the DE and ME content of the diet.

**Table 5:** Correlation coefficients between dietary fiber and available energy of diets

**Items**	**TDF**	**DE**	**ME**
Total dietary fiber ^a^, %	1.00		
Digestible energy, MJ/kg	- 0.92	1.00	
Metabolizable energy, MJ/kg	- 0.83	0.97	1.00

^a^ TDF = Total dietary fiber, DE = digestible energy, ME = metabolizable energy.

## Discussion

The level of fiber in a pig’s diet is considered an important factor affecting palatability and feed intake although pigs can tolerate relatively high levels of fiber [21]. High fiber levels in diets can decrease the voluntary feed intake of the animals as a consequence of gut fill, compromising the energy intake of pigs [22]. In the present study, the voluntary feed intake of pigs was lower than the full allowance during the trial period when the inclusion of sugar beet pulp was higher than 35.0% or TDF was higher than 24.8%. Likewise, Anguita et al. (2007) also reported that the inclusion of sugar beet pulp decreased voluntary feed intake of pigs more than other less digestible ingredients [23], probably as a result of the higher amount of digesta and its water retention capacity when the diet contained sugar beet pulp.

Fiber-containing diets could increase fecal output [24]. Wilfart et al. (2007) reported that the output of fecal dry matter increased as TDF increased [12]. In the current study, the output of feces increased as the TDF level increased. The main reason for this included two aspects. One reason is mainly due to the fact that the moisture content of feces increased as the TDF level increased. Another reason is the TDF excretion increased because 50 to 60% of the dry matter excretion at the rectum was TDF [12]. Generally speaking, the increase in fecal output as TDF level increased in the diet was related to the water holding capacity of SDF and increases in fecal bulk of IDF [4].

Soluble fiber is usually susceptible to microbial degradation, thus increasing bacteria growth in the lower gut [25].The greater the amount of fiber in the diet, the greater the disappearance of fiber (g of fiber disappeared/kg of feed DM) after fermentation in growing pigs [26]. Bindelle et al. (2009) added sugar beet pulp at levels of 0, 10, 20 and 30% to a corn-soybean meal basal diet fed to growing pigs and found a linear increase in the digestibility of neutral detergent fiber [14]. In agreement with the report of Bindelle et al. (2009) [14], the digestibility of TDF, SDF and IDF increased as the TDF level increased in the present study. One reason for this observation may be that there are components in endogenous secretions that are analyzed as TDF (although they are not TDF). The influence of these components is reduced as more TDF is included in the diets, which is the reason for the increased values for ATTD as TDF concentrations in the diets. But this result is not in line with the report of Wilfart et al. (2007) [12], who reported that the digestibility of TDF is unaffected by TDF level of diets fed to growing pigs. The occurrence of different results about the fiber digestibility may be due to different fiber sources used in the two trials (wheat bran vs. sugar beet pulp).

The strong relationship between the ATTD of TDF and the ATTD of IDF but poor relationship between SDF in the current study is in agreement with Urriola et al. (2010) [5], who reported that there was a strong relationship between the ATTD of TDF and the ATTD of IDF in distillers dried grains with solubles but a poor relationship between the ATTD of TDF and the ATTD of SDF. The poor relationship between the ATTD of TDF and the ATTD of SDF is due to the fact that most of the fiber in distillers dried grains with solubles is insoluble [27]. In this study, although the main component of sugar beet pulp is fiber, and the fiber is about 1/3 soluble and 2/3 insoluble (Table 1), the ATTD of SDF was higher than 92.0%. In other words, most of SDF in sugar beet pulp was fermented in the hindgut. Therefore, there is a poor relationship between the ATTD of TDF and the ATTD of SDF in the present study.

Fiber may improve intestinal health because it is necessary for the stimulation of the intestinal compartments [28], and it is usually associated with a reduction of potentially harmful products from protein fermentation [29]. However, the inclusion of fiber in the diet offered to pigs results in reductions in foregut and whole-tract digestibility of dry matter [27] leading to a lower absorption of nutrients and energy. The digestibility of nutrients in pig diets has been shown to be related to the origin and content of dietary fiber [30]. In the present study, inclusion of sugar beet pulp in a casein-rice starch basal diet increased the concentration of TDF, SDF and IDF in the diet. The ATTD of dry matter, crude protein and energy were negatively correlated as sugar beet pulp level increased from 15.0 to 55.0%. The lower ATTD of crude protein can be explained by increased endogenous secretions, or by decreased hydrolysis and absorption of nutrients, or both [12]. Part of the endogenous nitrogen loss was the bacterial nitrogen in the feces. It has been reported that 60 to 90% of fecal nitrogen was of bacterial origin [31]. Bindelle et al. (2009) examined the effect of dietary fiber on bacterial protein synthesis and reported a linear increase of bacterial nitrogen incorporation with graded levels of sugar beet pulp at levels of 10, 20, and 30%, respectively [14]. Similar to the report of Bindelle et al. (2009) [14], the excretion of fecal nitrogen increased when the TDF level increased. This may be the main reason for the decrease in digestibility of crude protein. Just et al. (1984) reported that dietary fiber concentration could account for about 70% of the variation in energy digestibility in diets [32]. Previous studies showed that the digestibility of gross energy decreased with an increase of TDF in the diet [12-14]. In agreement with previous reports, it was found that the digestibility of gross energy decreased when TDF level increased in the diet in this study. Castiglia-Delavaud et al. (1998) reported that about 35.0% of the fermented sugar beet non-starch polysaccharide energy appeared as fecal bacteria energy [33].

Evaluation of the available energy content of pig feeds is usually based on their DE or ME content [3]. It was found that a high fiber content is responsible for adverse effects on the digestible energy content of feeds for pigs [34]. A similar result was found in the present study that the content of TDF in the diet was negatively related to the DE and ME content of the diet. In addition, in agreement with the work of Noblet (2006) [3], who reported that the ratio of ME to DE of complete feeds is approximately 0.96 while in this study the ratio of ME to DE was 0.97.

## Conclusions

It is concluded that the digestibility of dry matter, gross energy, and crude protein in diets were negatively affected by the level of sugar beet pulp, which ranged from 15.0 to 55.0%, but the digestibility of SDF and IDF increased with the increase of TDF. There was a strong relationship between the ATTD of TDF and the ATTD of IDF, but the relationship between ATTD of TDF and the ATTD of SDF was poor.

## Abbreviations

TDF: Total dietary fiber; SDF: Soluble dietary fiber; IDF: Insoluble dietary fiber; ATTD: Apparent total tract digestibility; DDGS: Distillers dried grains with soluble; DE: Digestible energy; ME: Metabolizable energy.

## Competing interests

The authors declare that they have no competing interests.

## Author’s contributions

WJZ carried out the experiment trial, performed the statistics and drafted the manuscript. DFL and LL participated in design of the study. JJZ and QWD participated in animal trial. WJY participated sample analysis. LYZ conceived the study, and participated in its design and coordination. All authors read and approved the final manuscript.
